# Household

**Published:** 1889-07

**Authors:** 


### HOUSEHOLD.

FROZEN COFFEE.—Take two quarts of fresh filtered, or spring water, if obtainable, bring it to the boil, then add half a pound of the best old government Java coffee, roasted and ground; stir well together, cover and set aside on the range to infuse. Stir occasionally for the first ten minutes, then let it stand in a warm place till well settled. Now strain the coffee clear through a fine muslin cloth and add water to make two quarts; dissolve one pound of pulverized sugar in it, and set aside to cool; then pour it into the freezer, add the whites of two eggs, and freeze the mixture to a softish texture. This *frappe* is generally served in high glasses. On the continent of Europe this ice is called *cafe mousseux*, also *cafe frappe a la glace*. The fourth part of a vanilla bean is also sometimes infused in the coffee when making it, and tends to heighten the aroma of the coffee. Some persons also add half a pint of rich cream to it before freezing. The addition of these, however, are matters of taste and fancy.

WALNUT CAKE.—Three eggs, one and one-half cups of sugar, one-half cup of water, two cups of flour mixed with two teaspoonfuls of baking powder. Beat eggs and sugar thoroughly five minutes, add water and flour alternately. Bake in two oblong tins in a quick oven.

GEMS.—Take two cups of whole wheat flour (graham can be used in the place of whole wheat, if preferred), and one and a half cups of cold milk, the colder the milk the better. Take an egg, and beat the yolk and white separately, beating the yolk first, because that will not hold so much air as the white. The reason why we use an egg is that the albumen is capable of holding so much air. These gems can be made just as well without an egg after one is accustomed to the process, but at first it serves to give confidence. First add the beaten yolk of the egg to the milk, and stir vigorously, so as to mix thoroughly. Add flour to the milk and egg, a little at a time, beating all the while, up and down, not stirring the spoon round and round. This is done to incorporate as much air as possible. Add the flour little by little in this way, and the batter will become foamy and light ; but beat no longer than is required to mix in the whole of the flour smoothly. Lastly, add the white of the egg, stirring that round and round and rather lightly, for we want to keep in the air it contains. Put the batter into a hot gem-pan, and bake quickly in a hot oven. Indeed, the entire operation should be performed as quickly as possible. The gem-pans need to be kept thoroughly clean and well scoured, and then they will seldom require oiling.

To prevent flat-irons from rusting, wipe with a cloth dampened with kerosene.

STONE or marble hearths should be treated with pumice-stone and soap, rinsing carefully afterward.

SEVERAL tablespoonfuls of ammonia should be added to the water with which blinds and all dark paint are washed. When dry, rub with kerosene oil.

TOMATO FRITTERS. — One quart stewed tomatoes, one teaspoonful soda, Mix with enough flour to make a batter, as for griddle cakes. Drop the batter, a spoonful at a time, in hot oil or butter and fry.

